# The Biological Activities of Sesterterpenoid-Type Ophiobolins

**DOI:** 10.3390/md15070229

**Published:** 2017-07-18

**Authors:** Wei Tian, Zixin Deng, Kui Hong

**Affiliations:** Key Laboratory of Combinatorial Biosynthesis and Drug Discovery, Ministry of Education, and Wuhan University School of Pharmaceutical Sciences, Wuhan University, Wuhan 430071, China; twwtss@163.com (W.T.); zxdeng@whu.edu.cn (Z.D.)

**Keywords:** ophiobolins, source, bioactivities, structure–activity relationship, mechanisms

## Abstract

Ophiobolins (Ophs) are a group of tricarbocyclic sesterterpenoids whose structures contain a tricyclic 5-8-5 carbotricyclic skeleton. Thus far, 49 natural Ophs have been reported and assigned into A–W subgroups in order of discovery. While these sesterterpenoids were first characterized as highly effective phytotoxins, later investigations demonstrated that they display a broad spectrum of biological and pharmacological characteristics such as phytotoxic, antimicrobial, nematocidal, cytotoxic, anti-influenza and inflammation-promoting activities. These bioactive molecules are promising drug candidates due to the developments of their anti-proliferative activities against a vast number of cancer cell lines, multidrug resistance (MDR) cells and cancer stem cells (CSCs). Despite numerous studies on the biological functions of Ophs, their pharmacological mechanism still requires further research. This review summarizes the chemical structures, sources, and biological activities of the oph family and discusses its mechanisms and structure–activity relationship to lay the foundation for the future developments and applications of these promising molecules.

## 1. Introduction

Sesterterpenoids are a group of compounds with C_25_ carbon frameworks derived from five isoprene units, and exhibit a variety of biological activities including cytotoxic and antimicrobial functions [1,2,3]. They have been found from widespread sources including fungi, bacteria, plants, insects and marine organisms [4,5]. Marine organisms represent a broad source of potential sesterterpenoids. For example, genus *Phyllospongia*, one of the most common marine sponges, has been shown to be a prolific producer of various scalarane sesterterpenoids [6], and marine-derived fungi belong to the genus of *Aspergillus* produced sesterterpenoid ophiobolins [7].

**Figure 1:**
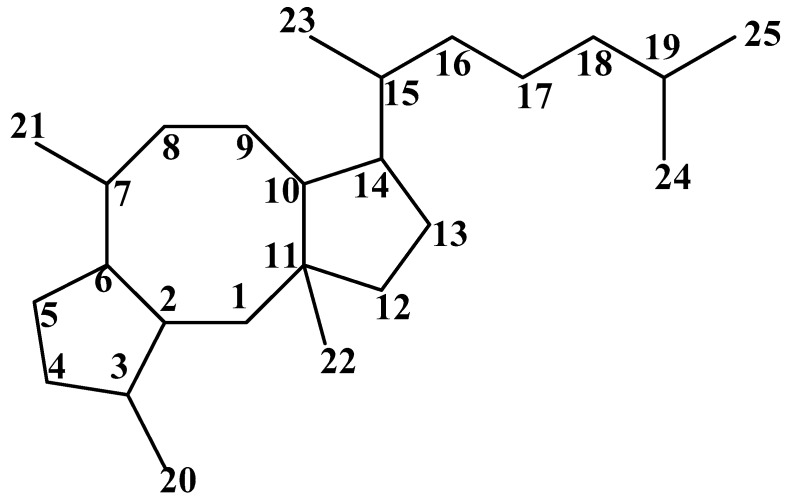
Carbon skeleton of Ophs.

**Figure 2:**
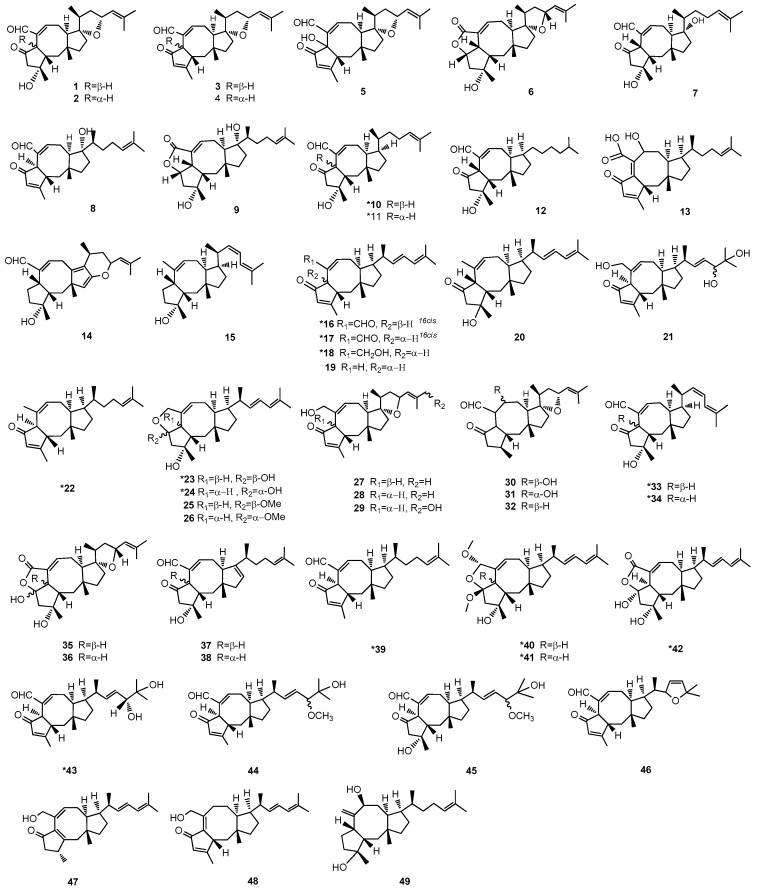
Structures of Ophs. * The compounds from marine-derived fungi. All assigned stereocenters of the structures of Ophs have been drawn in Figure 2. However, there are still some undepicted stereochemistry of structures in Figure 2 because the stereochemistry in the original literature was not supplied.

Ophiobolins (Ophs) are a class of sesterpenoids and characterized by the structure of a tricyclic 5-8-5 ring system derived from head to tail linkages of five isoprene units (Figure 1). These small molecules, ranged in molecular weight from 338 to 432, are produced by fungi mainly belong to the genus *Bipolaris* and *Aspergillus*. The first member of this family named ophiobolin A (compound **1**, Figure 2) was isolated from the pathogenic plant fungus *Ophiobolus miyabeanus* by Nakamura and Ishibashi in 1958 [8]. Numerous additional analogs have been reported since then. Here, we summarize 49 natural products of Ophs, which are assigned into 23 subgroups (A–W).

There are enormous potential applications of Ophs in different fields due to their wide biological effects. Initially, the researchers focused on the investigations of phytotoxic properties of Ophs, which are produced by pathogenic fungi attacking agricultural crops [9,10,11,12,13,14,15]. Later, Ophs have been proven to possess a broad spectrum of other biological properties, such as antimicrobial [15,16], nematocidal [17,18,19] and cytotoxic activities [1,5,9]. Additionally, MDR reversal effects of Ophs on multiple tumor cells with a low concentration were proven by both in vitro and in vivo experiments [20,21]. Furthermore, Ophs also possess anti-influenza [22] and inflammation-promoting activities [23]. With their diverse pharmacological properties, Ophs are shown to be a group of promising drug candidates. Although a variety of potential biological activities of Ophs have been widely investigated, their molecular targets, the functional mechanisms, and structure–activity relationships are still obscure. After Au et al. (2000) reviewed the biology of 23 Ophs [9], 26 more Ophs have been reported/added to the family. In this review, we summarize the chemical structures, sources, and biological properties of 49 reported Ophs. Furthermore, we will focus on the cytotoxic properties, mechanism of action, and structure–activity relationship of Ophs to demonstrate their pervasiveness and importance in drug discovery.

## 2. Chemical Structure and Source of Ophs

These small molecules are characterized by a core structure of 5-8-5 tricyclic carbon skeletons (Figure 1) and assigned into A–W subgroups. All reported Ophs, numbered from 1–49, are shown in Figure 2. The original names of the biological sources of Ophs are listed in Table 1.

With many structurally similar analogs, Ophs family needed an approach to distinguish between them [24,25]. Kildgaard et al. (2014) reported that Ophs can be dereplicated accurately by using ultra-high performance liquid chromatography–diode array detection–quadrupole time of flight mass spectrometry (UHPLC-DAD-QTOFMS) with a tandem high resolution MS (MS/HRMS) Library and eight Ophs were available as standards and included in the library at present [25].

In the earlier studies, different research groups adopted different taxonomical nomenclatures for the same pathogenic fungi, which caused confusions. Therefore, some of the fungi have been reclassified. For example, *Drechslera oryzae* and *Helminthosporium oryzae* have been reclassified in *Bipolaris oryzae*.

The majority of Ophs were discovered from the fungi genera of *Bipolaris* and *Aspergillus*. For example, genus *Bipolaris* produces ophiobolins A–B, I–J, and L–M, while genus *Aspergillus* produces ophiobolins C, F–H, K, N, and U–W (Table 1). Marine-derived fungi belong to *Aspergillus* produce various Ophs. For example, *Aspergillus* sp. (Taxonomy ID: 5065) produces ophiobolins G, H, K and O [4] (Table 1); and *Emericella variecolor* (reclassified in *Aspergillus stellatu*, Taxonomy ID: 1549217) produces ophiobolins C, G, H, K and N [26,27] (Table 1). The other genera including *Drechslera* [28], *Ulocladium* [29], *Penicillium* [30], *Mollisia* [31], and *Cephalosporium* [32], were producers of a part of the discovered structures. Moreover, ophiobolin A lactone and ophiobolin B lactone were transformed from ophiobolin A by the bacteria *Polyangium cellulosum* and *Pseudomonas aeruginosa*, respectively [30].

It is not yet known whether the similarities in structures of Ophs are drastically associated with genus. The Ophs (compounds **1**–**5**, **27**–**32**, **35**, and **36**) with furan structure at C14-17 are majorly produced by *Bipolaris*, while the Ophs without this structure are majorly produced by *Aspergillus*. On the other hand, *Cochliobolus heterostrophus*, which was later classified to *Bipolaris*, can produce both structures with (compounds **1**–**4**, **27**, and **35**) or without (compounds **7**, **8**, **10**–**12**, **15**, **23**, **33**, **34**, **37**, and **38**) the furan ring.

It is worth noting that the biosynthetic production of Ophs is influenced by the different culture conditions. For example, *Drechslera gigantean* (Taxonomy ID: 1937655), a global fungal plant pathogen, was able to produce **1**, **2**, **4**, and **27** in liquid cultures. However, in solid conditions, it produced **7**, **14**, **30**, and **31** [26]. While all the compounds produced from liquid culture and some compounds from solid culture have the furan structure, **7** and **14** did not have the ring. Before confirmation of the genus position of *Cochliobolus heterostrophus* from different reports, the correlation between the Ophs production and the taxonomic position of their source strains or their culture conditions is still uncertain.

## 3. Phytotoxic Activities

Early research considered Ophs as the metabolites of pathogenic fungi, which attacked various crops by inducing brown spot lesions on the leaves [9]. These crops included monocotyledons and dicotyledons species, especially grass weeds [12]. Furthermore, compound **1** inhibited the growth of Tobacco Bright Yellow-2 (TBY-2) at concentrations of 2–5 μM [21,50]. Table 2 lists the phytotoxic activities of Ophs on 12 tested plants.

Compound **1** was proven to be phytotoxic to almost all of the tested plants, even at the lowest concentration. Compound **1** gave necrotic legions on cabbage and corn while **2**, **3**, **4**, **7**, and **27** did not (Table 2) [13,14,15]. Ophs (**1**, **2**, **3**, **4**, **7**, **27**) showed phytotoxic properties by causing necrosis on the plants except tobacco [15]. Furthermore, the Ophs exhibited other phytotoxic effects: **1** was effective in reducing sheath blight incidence under field conditions [51], and both **16** and **23** inhibited growth of etiolated wheat coleoptile at 10 μM [44]. Furthermore, while Xiao et al. (1991) had illustrated that **27** (1.2 × 10^3^ μM) was inactive to corn leaves [14], Sugawara et al. (1987) and Kim et al. (1999) demonstrated it was active to corn leaves at a lower concentration (1.0 × 10^3^ μM) [13,15].

The ophiobolin A-series **1**, **2**, and **3** appeared to be more active on the tested plants than other Ophs, but **4** displayed low activity at the same concentration (Table 2). Compounds **1** and **3** were β-H isomers of **2** and **4** at C-6, respectively. Comparing phytotoxic effects between the two groups of isomers, β-H (C-6) of compounds enhanced the action on barley, corn, goose grass, and velvet-leaf [14,15]. However, β-H (C-6) of **1** suppressed the action on cocklebur and Johnson grass, and there was no influence on sorghum and soybean [13,15] (Table 2). However, β-H (C-6) of **3** improved activity on cocklebur, rice and soybean [15] (Table 2). Therefore, the structure-phytotoxic activity of stereochemistry at C6 was obscure, as β-H (C-6) did not always elevate phytotoxic effect.

The structures of **3** and **4** lacked a hydroxy group at C-3 on the basis of **1** and **2**, respectively. The hydroxy (C-3) of **1** improved the inhibition against barley and cabbage, reduced the activity on cocklebur and tested plants, while the activity on the remaining tested plants was unchanged (Table 2) [13,14,15]. The hydroxy (C-3) of **2** elevated phytotoxic effects on all the tested plants except grain sorghum and velvet-leaf, whose growth had no distinction between **2** and **4** (Table 2) [13,15]. At this moment, it is impossible to determine whether the hydroxy group at C-3 of Ophs improved their phytotoxic activity or not. For hydroxyl at C-14, there is no difference between the activities of **7** and **10** on corn, Johnson grass, and sorghum (Table 2) [13]. However, comparing with **1**, when there was no hydroxy group at C-3 and α-H existed at C-6 in **4**, the phytotoxic action was significantly decreased in all the test plants [13,14,15] except goose grass (Table 2) [15].

Sugawara et al. (1987) demonstrated that compound **1** was much more phytotoxic than **10** against corn, Johnson grass, and sorghum, which suggested that the lack of the configuration of the tetrahydrofuran ring between C14 to C17 possibly reduced phytotoxicities (Table 2) [13]. The structural difference between **1** and **27** was that hydroxy (C3) and aldehyde (C21) attached to **1**, double bond (C3) and hydroxy (C21) attached to **27**. Compound **1** visibly elevated the activities on all the experimental plants except cocklebur, goose grass, and velvet-leaf [15], whose growth had no difference between them (Table 2) [13,14,15]. The carbonyl group (C21) of **3** significantly improved the phytotoxic effect on all the tested plants except cabbage [14] and goose grass [15], the growth of which had no difference (Table 2) [13,14,15].

Besides the phytotoxic effects displayed in Table 2, molecules **1**, **2** and **4** were proven to be phytotoxic to several monocotyledon and dicotyledon weeds [12]. The structure–activity relationship was similar to the above characterizations. β-H (C-6) did not always enhance phytotoxic effect when comparing **1** with **2**. Whereas **1** displayed strong activities on nearly all the tested weeds in comparison with **4**, the possible structural features for reducing phytotoxic activities could be a combination of the lack of the hydroxy group at C-3 and the stereochemistry of hydrogen atom at C-6 (from β-H to α-H) [12].

Ophs produce phytotoxic activities in plants through multiple mechanisms of action. Early studies have demonstrated that both **1** and **7** could inhibit the germination and growth of *Oryzo sativa* seeds [11], infect rice leaves, and cause leaf brown spot through reducing the photosynthetic leaf area and the efficient use of nutrients [52]. It is observed that **1** have stimulated the net leakage of electrolytes from roots, leaves, and coleoptiles of rice plants and loss of β-cyanin from beetroot tissue at 250 μM [53]. Furthermore, the molecule induced a loss of osmotic pressure in the guard cells, ultimately resulted in stomatal closure and decreases of *g*_s_ [54], and caused loss of electrolytes and glucose from maize and carrot roots [10]. Treat tissues with **1** effectively inhibited the uptake of exogenous ^32^PO_4_ and 2-deoxyglucose at 25 and 250 μM, respectively [10,53]. In addition, **7** exhibited inhibitory effects of proton extrusion from maize coleoptiles [39]. The release of electrolytes could explain that the phytotoxicity of Ophs, in stimulating intracellular potassium, phosphate, glucose, carbohydrates, amino acids, and proteins leakage, influenced the transport processes of plasma membrane systems [10,39,53]. Later, it has been demonstrated that the activity of **1** associating with calmodulin played a critical role in the development of cell cycle [55]. Moreover, lysine 75 was the primary inhibitory site for **1**, other amino acids including lysine 75, 77, and 148 acted as the binding sites of calmodulin antagonism [56,57]. Furthermore, compound **1** performed the inhibitory of TBY-2 through arresting the cell cycle and altering the intracellular partitioning of glutathione between nuclei and cytoplasm [21,50].

## 4. Antimicrobial Activities

### 4.1. Antibacterial Activity

Ophs exhibited antibacterial actions against *S. aureus*, *M. intracellulare, B. subtilis*, MRSA, BCG, and *M. smegmatis*, but almost all the tested Ophs did not possess activities against *E. coli* (Table 3), including **1**, **7**, **16**, **23**, **24**, **27**, **35**, **36**, and **47** [30,43,44].

Though compounds **16** and **17**, **23** and **24**, **33** and **34**, and **35** and **36** were β-H and α-H isomers at C6, respectively, it was not obvious that the stereochemistry of H atom at C-6 affected the antibacterial activities of Ophs (Table 3) [27,29,30,43,44]. Furthermore, compound **5** displayed a significant enhancement of antibacterial effects against *B. subtilis*, MRSA, and BCG, when compared with **3** and **4**, which illustrated that the hydroxyl group at C-6 obviously improved the antibacterial actions [38].

Although Ophs showed weak antibacterial effect, they acted on both gram negative and positive bacteria. Ophs prevented bacterial biofilm formation. Molecules **17**, **33**, and **34** inhibited biofilm formation of *M. smegmatis* with minimum inhibitory concentration values of 17, 4.1, and 65 μM, respectively. Molecule **33** also inhibited the biofilm formation of *M*. *bovis* BCG at 8.2 μM [27]. Since the biofilms protected the microbes against the action of most antibiotics, the activity of Ophs against biofilm formation provided a promising therapeutic strategy against biofilm infections.

### 4.2. Antifungal Activity

At 60 μM concentration, compounds **1**, **7**, **27**, and **36** strongly suppressed the growth of *T. mentagrophytes*, but there was no significant inhibition on *C. neoformans* and *C. albicans* [30]. In addition, compound **1** was proven to restrain the growth of numerous fungi such as *Gloeosporium*, *Glomerella*, *Corticium*, *Macrosporium*, and *Trichophyton* species [8]. Moreover, it also exerted activity against *Aspergillus flavus*, *Trichophyton mentagrophytes, Torulopsis cremoris*, and *Torulopsis petrophilum*, with minimum inhibitory concentration (MIC) values of 60, 31, 0.5, and 0.5 μM, respectively [30].

Apart from the above species, the antifungal properties of **1** and **7** on 17 different zygomycetes fungi were characterized at the MIC values range of 8–125 μM. Crucially, the active molecule **1** displayed fungicide effect against *Rhizopus oryzae* and *Rhizopus stolonifer* at 15 μM, while **7** only showed fungistatic effect at a higher concentration of 60 μM [57], which demonstrated that lack of the configuration of the tetrahydrofuran ring between C14 to C17 possibly exhibited less antifungal activities in genus *Rhizopus*.

Furthermore, compound **1** significantly inhibited the germination of sporangiospore of *Mucor circinelloides* and caused morphological changes. The degenerated, thick or swollen cells, septa and damaged cell cytoplasm effusions were observed [57].

## 5. Nematocidal and Trypanocidal Activities

Ophs also presented nematocidal and trypanocidal activities (Table 4). Compound **10** displayed the best nematocidal activities against the *Caenorhabditis elegans* (*C. elegans*) at 5 μM (LD_50_ value) than other tested Ophs (**11**, **12**, **33**, **34**, **37**, **38**) in Table 4 [18]. Moreover, **37** and **38** also exhibited trypanocidal activities against *Trypanosoma cruzi* (*T. cruzi*) [19]. Importantly, **11**, **34**, and **38** were the α-H isomers of **10**, **33**, and **34** at C6, respectively. Nematocidal activity against the *C. elegans* of 6-epi isomers obviously reduced a lot, which indicated that the α-H at C6 suppressed the nematocidal activity. However, the stereochemistry of the hydrogen atom at C-6 did not influence the trypanocidal activity against *T. cruzi* (Table 4). Comparing to **10**, the olefinic bonds at C16-C17 of compound **33** and the olefinic bonds at C13-C14 of compound **37** exhibited nematocidal activity against the *C. elegans* at higher concentration.

Investigations illustrated that the nematocidal activities of Ophs were associated with their affinity to the binding site of ivermectin (a widely used insect repellant modulate glutamtate-gated chloride channel) and with their cytotoxic actions to fibroblast host cells [18,44].

## 6. Cytotoxic Activities

### 6.1. Cytotoxic Activity In Vitro

Mounting evidence confirmed broad but also selective cytotoxic activities of Ophs on multiple types of mammalian cells. Table 5 lists the IC_50_ values of Ophs on various cell lines, as collected from the literature. Though the results from different reports are not completely comparable, we attempted to gather some useful information based on the comparable data.

From 1999 to 2016, among the 49 Ophs, more than half (*n* = 26) of the molecules were reported for their cytotoxic activity. Compound **1** was the most widely studied molecule in the Ophs family. It displayed growth inhibition against 26 human cancer cells, three other mammalian somatic cells, two human normal cells (HUVEC and hPBMC), one MDR cell (LLC-GAS-COL150), and one murine cancer cell (P388) (Table 5). The IC_50_ values of **1** against 33 cell lines ranged 1 × 10^−3^ − 77 μM, but most of the values (30 out of 33) were lower than 10 μM. Though these results came from different reports, it is evident that **1** is the most cytotoxic in the Ophs family on the tested cells (Table 5). Molecule **1** has been evaluated on 60 cell lines in the NCI panel, where it registered an average GI_50_ value of 70 nM [67]. Bury et al. (2013) [21] revealed that slowly proliferating cells were less sensitive to the growth-inhibitory effects than the highly proliferative ones induced by **1**. Molecule **33** was the second most tested molecule, which inhibited the growth of 11 cancer cells at IC_50_ range of 4 × 10^−3^ to 0.65 μM. It also shows the potential to be applied in cancer therapy.

A total of 41 cells have been tested with Ophs molecules. The human normal cell line (HUVEC) was more sensitive to compound **1** at IC_50_ of 0.08 μM, comparing to **27** at IC_50_ > 1 μM. It was insensitive to **40** at IC_50_ of 85 ± 12 μM, from different reports (Table 5).

Concerning the inhibition of proliferation of haematological cancer cells, the lowest IC_50_ record of Ophs cytotoxic activity is 1 × 10^−3^ μM of **1** on CLL, while the highest is 106 μM of **23** on P388 cell except the inactive ones (Table 5). CLL was the most sensitive one but was also selective to different Ophs structures. Ophs **1**, **7**, **10**, and **33** displayed significant cytotoxic activities on CLL cell at low nanomolar concentration, while compounds **3**, **4**, **17**, **23**, **34**, and **39** were inactive on CLL cell [24]. K562 cell was more sensitive to compound **5** than to **3** and **4** [38]. Molecule **1** inhibited haematological cancer cells Jurkat, MM1R, RPMI8226, and U266B1 at low dose [61]. Furthermore, **1** was proven to exhibit cytotoxic action against the mouse leukemia L1210 cell in a concentration-dependent manner from 0.01 to 1 μM [60]. Mouse leukemia cell P388 was most sensitive to **40** among the other molecules of **16**, **23**, **33**, **34**, and **41**, at IC_50_ value of 4.7 μM. However, the IC_50_ values were 0.06 μM by **1** [35] and 0.51 μM by **33** [26] from the other reports. None of the compounds **18**, **21**, **24**, **25**, and **26** reduced survival rate of murine lymphoma L5178Y cell by more than 10–20%, comparing to the controls, even at a high concentration of 27, 25, 26, 25, and 25 μM, respectively [45].

Glioblastoma multiforme cells (GBM) are the most lethal and common malignant human brain tumors that are resistant to apoptosis and not sensitive to the classical chemotherapies. The growth of three GBM cell lines (GL19, T98G, U373-MG) was inhibited by molecule **1** at the IC_50_ values of 3.7, 1.9, and 0.87 μM, respectively [62]. Other reports indicated that **1** decreased the proliferation of Hs683 and U-87-MG cells at IC_50_ values of 0.62 μM [21] and 3.8 μM [64], respectively (Table 5). In addition, Ophs **10**, **11**, **16**, **17**, **23**, **33**, **34**, and **39** displayed cytotoxic effects against neuroblastoma cell (Neuro 2A), at concentration of 1–3 μM [26]. Furthermore, a potential therapy for neurodegenerative disorder diseases including Parkinson’s disease (PD) was reported for compound **5** that was identified to induce autophagy in GFP-LC3 stable HeLa cell at 10 μM. However, in this case, **1** did not present the same activity as **5**, while it was tested in the same experiments [68].

One attractive feature of Ophs, discovered recently, is their potential application on MDR cancer cells. Compound **1** was found to inhibit LLC-GA5-COL150 cells transfected with human MDR1 cDNA encoding P-gp at IC_50_ value around 7.9 μM [65]. It also displayed promising effects on MDR cancer cells, including HL60 cells resistant to adriamycin, vincristine, and mitoxantrone, ovarian carcinoma cell line resistant to cisplatin, small lung carcinoma cell line GLC4 resistant to adriamycin, and the human colon cancer HCT116 p53 (-/-) delete clone [21]. In addition, compound **40** could reverse the adriamycin resistance of MCF-7/ADR [20].

CD44^+^/CD24^−^ phenotype, which has been associated with stem cell-like characteristics, could be the specific markers of CSCs [69,70]. Molecules **1** and **7** possessed a highly potent CSC inhibitory activity to MDA-MB-231 breast cancer cell line and displayed similar effects in reducing the percentage of CD44^+^/CD24^−^ breast CSC subpopulation at 1 μM. It illustrated that the other breast cancer stem cells such as MDA-MB-436 and Hs578T, which contained CD44^+^/CD24^−^ population, also naturally established the inhibitory effect. It was confirmed that **1** significantly inhibited the mammosphere formation efficiency of MDA-MB-231, MDA-MB-436, Hs578T, and MCF7 grown in non-adherent conditions [71].

### 6.2. Cytotoxic Activity In Vivo

Ophs not only possessed inhibition in vitro cells, but also reduced tumor in vivo animal models. Molecule **1** was active in both mouse GBM [67] and melanoma [21] models, while **40** was effective in mouse breast model, either alone [66] or in combination with adriamycin (ADM) [20].

In vivo mouse GBM model, compound **1** significantly enhanced the survival rate of mice in bearing orthotopic U251-LUC tumors at 10 mg/kg (intraperitoneal administration). It also exhibited the capability of crossing the blood-brain barrier (BBB) [67]. In addition, **1** significantly improved the survival period of B16F10 melanoma-bearing mice at the dose of 10 mg/kg (intraperitoneal administration) [21].

Compound **36** expressed significantly reduction in tumor size in MCF-7 breast-bearing nude mice, with the inhibitory rates of 35%, 46%, and 69% at the 50th day of 5, 10, and 20 mg/kg (subcutaneous administration), respectively. The inhibition rate of 69% was nearly equivalent to the positive control group (73%) [66]. At the dose of 5 mg/kg, in the same model, combined treatment with **40** and ADM, the tumor inhibition rate in MCF-7 breast-bearing nude mice reached 71 ± 5.6%, while the inhibition rates were 46 ± 5.2% and 23 ± 3.8% of ADM and **40** alone, respectively [20].

### 6.3. Structure–Cytotoxic Activity Relationship

The structure–cytotoxic activity relationship of Ophs is complex. It is hard to establish the correlation between the structure and the cytotoxic activity at present. We will discuss the structural characteristics of Ophs frequently occurred in the following circumstances: (1) the stereochemistry of the hydrogen atom at C-6 position; (2) the hydroxyl group attached to C3, C6 and C14 position; (3) the position of tetrahydrofuran ring including tetrahydrofuran ring between C14 to C17 and C5 to C21; and (4) other structure–cytotoxic activity relationships.

#### 6.3.1. Stereochemistry at C6

Molecules **1** and **2**, **3** and **4**, **33** and **34**, **40** and **41** were pairs of isomers with β and α-hydrogen atom at C-6, respectively. Comparing **1** with **2**, α-H enhanced about 4.4 and 3.5 times of cytotoxic activity in Hela and KB cells, respectively (Table 5) [33]. When comparing **3** with **4**, the stereochemistry at C6 practically did not change the cytotoxic activity in CLL [24], K562 [38] and HepG2 [38] cells (Table 5). However, when comparing **33** with **34**, β-H significantly elevated cytotoxic effect in CLL cells [24], and the enhancement of activity in P388 [7] and TK-10 [19] cells was approximately 2 and 6 folds, respectively (Table 5). Comparing **40** with **41**, β-H also slightly improved about 2 times of cytotoxic activity in murine P388 cells (Table 5) [35]. Overall, no firm conclusions can be drawn, as improved cytotoxic activity of β-hydrogen atom of Ophs was not consistent.

#### 6.3.2. Hydroxy Group at C3, C6, C14

The most useful features for distinguishing molecule pairs **1** and **3**, **16** and **33**, **17** and **34**, and **44** and **45** were the functional group at C3. The former of the pair compounds possessed the hydroxy group while the latter carried double bond. The cytotoxic effects of the hydroxy group at C3 of **1** were 6.3 times lower, when comparing with **3** in Hela and KB cells [33]. However, the decreased effect was slighter when comparing **16** to **33** in P388 cells [7], **17** to **34** in HepG2 and KB cells [29], and **44** to **45** in HepG2 and KB cells [29], respectively (Table 5). In CLL cells, hydroxy group at C3 visibly improved the cytotoxic effect when comparing **1** to **3** (Table 5) [24]. As far as the evidence is concerned, the hydroxy group at C3 was less efficient than the double bond between C3 and C4 under more extensive circumstance. On the other hand, the fact that **5** with hydroxy group at C6 was about 8–9 times more efficient than **3** and **4** in inhibiting the proliferation of both K562 and HepG2 cells (Table 5), suggesting a positive role of the hydroxy group at C6 [38]. Najumudeen et al. (2016) found that at the same concentration, when **10** was almost inactive, **7** highly inhibited CSC MDA-MB-231 breast cancer cell line, which indicated that the hydroxy group at C14 of **7** enhanced the anti-CSC effects [71]. However, Bladt et al. (2013) reported that the hydroxy group at C14 of **7** was about 4 times less efficient than **10** in CLL cells [24]. More investigations are needed to prove whether the hydroxyl group attached to the C6 or C14 positions of Ophs is able to enhance the cytotoxic activities.

#### 6.3.3. Tetrahydrofuran Ring

Although 5-8-5-ring system was the mutual structure of the Ophs family, there was another ring exhibited in many Ophs. The tetrahydrofuran ring between C14 to C17 is present in compound **1**, while tetrahydrofuran ring between C5 to C21 appears in **23**, **40**, and **41**. The position of tetrahydrofuran ring influenced the activity of Ophs.

The major functional group difference between molecules **1** and **10** was the absence of the tetrahydrofuran ring (C14–C17) in the latter, which also presented in **1** and **33**, as mentioned above. Molecule **1** enhanced inhibitions on CLL cells eight and four folds, in comparison with **10** and **33** (Table 5) [24], respectively. It demonstrated that the tetrahydrofuran ring between C14 to C17 displayed effect on improving activity. However, Najumudeen et al. (2016) reported that molecule **1** exhibited a highly potent CSC inhibitory activity to MDA-MB-231 breast cancer cell line, while **10** was almost inactive at the same concentration, which indicated that the tetrahydrofuran ring between C14 to C17 elevated the anti-CSC activities [71]. The major functional group difference between molecules **23** and **33** was the absence of the tetrahydrofuran ring between C5 and C21 with β-hydroxy group at C5 of the latter. When comparing **23** with **33**, tetrahydrofuran ring (C5–C21) of **23** significantly suppressed the activity in CLL cells (Table 5) [24]. Besides, as characterized above, **23** also decreased about 7.4 times effective in P388 (Table 5) [7]. Nevertheless, the tetrahydrofuran ring between C5 to C21 also appeared in **40** and **41**, which revealed that the two oxygenated methyls at C5 and C21 possibly enhanced the inhibitory activity, while the β-hydroxy group at C5 reduced it [7,35].

In conclusion, more evidence is required to determine whether the tetrahydrofuran ring between C14 to C17 elevated the cytotoxic activities of Ophs. Nonetheless, according to the literature, it is reported that tetrahydrofuran ring between C5 to C21 suppressed the cytotoxic activity.

#### 6.3.4. Other Structure–Cytotoxic Activity Relationship

The majority of cytotoxic Ophs possessed dicarbonyl in C5 and C21, while some of them exhibited hydroxyl at C-21. The distinctive molecules pairs were **3** and **27**, **17** and **18**, the former compounds possessed carbonyl at C21, while the latter possessed hydroxyl. The carbonyl group (C21) of **3** suppressed the cytotoxic effects approximately 40 and 5.3 folds in Hela and KB cells (Table 5) [33], respectively, comparing with **27**. However, when comparing between **17** and **18**, the cytotoxic activity of them was opposite. The carbonyl group (C21) of **17** elevated the activity about 6 and 2.9 times in HepG2 and KB cells (Table 5) [29], respectively.

The most structural difference between molecules **1** and **4** was the anhydration at C3 and the stereochemistry at C6. Molecule **1** displayed anti-proliferative effect against CLL cells while **4** showed inactivity, which illustrated that the combination of anhydration at C3 and α-H at C6 has visibly suppressed the anti-proliferative effects (Table 5) [24]. This structure–cytotoxic activity relationship was also significantly exhibited in A549, B16F10, Hs683, and SKMEL28 cells (Table 5) [21].

Compound **1** reduced the growth of both OVCAR3 cells and HUVECs at very low concentrations of 0.28 μM and 0.08 μM, respectively (Table 5). Compound **27** was about 250 times less potential than **1** in inhibiting the proliferation of OVCAR3 cells at 71 μM and displayed no effect against HUVECs at all, which revealed that the combination of a hydroxy group at C3 and an aldehyde at C21 of **1** significantly enhanced the activity than the double bond at C3 and the hydroxy group at C21 of **27** [58].

Besides the characterization above, Dasari et al. (2015) suggested that the carbonyl groups in C5 and C21 were the functional group of the cytotoxic action, since the reaction of **1** with primary amines [67]. A remarkable cytotoxicity decrease can be noticed in Table 5 for the compounds without this structure (**23**, **24**–**26)**. However, oxygenated methyls on C5 and C21 of **40** and **41** [7], and hydrogen on C5 of **42** and **44**, are capable of reversing the decrease (Table 5) [29].

### 6.4. Mechanism of Cytotoxic Activities on Cancer Cells

As molecule **1** presented broad cytotoxic activity, most of the Ophs cytotoxic mechanism studies were based on this compound. Generally, the study of cytotoxic mechanisms of Ophs was related to the identification of target proteins and pathways, and the observation of cell death phenotypes including apoptosis, paraptosis, and necrosis.

#### 6.4.1. Presumed Protein and Non-Protein Targets

Early study illustrated that the cytotoxicity of **1** was attributed to its covalent binding to calmodulin, and lysine 75 was discovered as the inhibitory site identified by site mutation. However, the Lys-75 mutants were only partially resistant to **1**, and the bovine brain calmodulin binding sites were different from those of plant cells [72]. Through the inactivation of calmodulin, **1** was established as the most potent compound among the tested molecules, which exerted its specific activity target K-ras 4B and suppressed the expression of the stemness marker Sox2 in four types of breast CSCs [71]. Furthermore, as a calmodulin inhibitor, **1** was found inhibiting cell migration, and rendering the cells to be sensitive to thapsigargin treatment that related to endoplasmic reticulum (ER) membrane protein Sec62. Sec62 is essential for cell migration and protecting tumor cells against thapsigargin-induced ER stress, which are both linked to cytosolic Ca^2+^. Sec62 protein levels are significantly increased in different tumors, including prostate, lung, and thyroid cancer [73]. A recent report by Morrison et al. (2017) claimed that **1** displayed different mechanisms of cell death in eight mammalian cells depending upon the cancer cell origin. We noticed that though most of the qualitative effects induced by **1** were various and non-correlated, the concentrations of free Ca^2+^ in the cytosol ([Ca^2+^] i) were increased in seven of the eight tested cell lines, with the only exception being Hela cell lines; and the IC_50_ growth inhibitory concentration of Hela cell lines was the highest among the eight cell lines [64]. Despite of the above evidences, it has been shown that there is no correlation between the mRNA expression levels of calmodulin and the toxicity of **1** [64]. However, it is certain that the mechanism of Ophs’ cytotoxicity has relation with Ca^2+^ implicated cell activities.

The inverse docking (INVDOCK) analysis indicated that **40** could bind to GSK3β. The cyclin D1 degradation and G1 phase arrest caused by **40** was abolished, while GSK3β was deleted using siRNA [66]. Though **1** [63] and **40** [20] were found targeting to MDR-related efflux pumps by inhibiting the P-gp expression. Clear non-selective inhibition were observed for **1** on both non drug resistant cancer cells and MDR cancer cells with over expression of P-glycoprotein, MRP-1, and LRP-1, and p53 (-/-) delete clone [21]. It is inferred that **1** does not directly react to these MDR symbol markers.

A non-protein target of phosphatidylethnolamine (PE) was proposed by Chidley et al. (2016). Based on a loss-of-function genetic screening in human haploid KBM7 cells, cytotoxicity of **1** was mitigated due to the reduction of PE by genetic inactivation of the de novo synthesis. Though **1** was proven to be not reacting with any of the three enzymes involving the de novo PE synthesis, it directly formed covalent pyrrole-containing cytotoxic adducts with the ethanolamine head group of PE. The authors hypothesized that the formation of PE-OPA adducts disrupted the lipid bilayer of human cells to induce cell death [74]. This non-protein target related to lipid membrane is useful to explain the broad bioactivities of Ophs since PE is ubiquitously found in the nature. On the other hand, the carbonyl groups in C5 and C21, which are the major functional groups reacted with the primary amine, were absent in some Ophs that presented cytotoxic activities such as **40**, **41**, and **42**; and the cytotoxic IC_50_ values were very different among the molecules that possess this structure such as **1**–**5**, **7**, **10**, **16**, **17**, **33**, **34**, **39** and **43**–**46** in Table 5. Further investigation should be carried out to clarify this difference.

#### 6.4.2. Pathways Related to Ophs Cytotoxicity

Signaling pathways of PI3K/mTOR, Ras/Raf/ERK, and CDK/RB are proven to be intersecting in the crucial cell processes including autophagy, apoptosis, and cellular homeostasis [75,76]. Bhatia et al. (2016) illustrated that compound **1** inhibited the generation of breast carcinoma MDA-MB-231 cells, involving the above three signaling pathways, through reducing phosphorylation level of ERK, S6, AKT, Cyclin D1, and phospho RB in a dose-dependent manner [61]. Yang et al. (2012) reported that **40** activated JNK (c-Jun NH2-terminal kinase), p38 MAPK (mitogen activated protein kinase), and ERK (extracellular signal-regulated kinase) pathways, and exhibited cell cycle arrest at G_0_/G_1_ phase [77]. Further investigation indicated that the arrest in the G_1_ phase was correlated with AKT/GSK3β/cyclin D1 signaling by reducing the phosphorylation level of the protein of AKT and GSK3β, and inducing down-regulation of cyclin D1 [66].

Autophagy is defined as an evolutionarily conserved catabolic pathway responsible for bulk degradation of intracellular components including miss-folding protein, protein aggregates, and damaged organelles. Compound **5** was found to cause autophagy in GFP-LC3 stable HeLa cells by the degradation of α-synuclein, induction of ROS and the activation of JNK signaling [68]. It is believed that targeting compensatory survival pathways in cancer by using a single pharmacological agent is an attractive anticancer strategy to overcome drug resistance resulted from the inhibition of single target protein or pathway [61]. On the other hand, the multi-targets function of Ophs suggests that some mechanism above cell level may exist.

#### 6.4.3. Cell Death Phenotypes

Apoptosis is a process of programmed cell death induced by multiple factors including activating caspases. The first discovery of **1** induced apoptosis was observed by Fujiware et al. in 2000. It was in a dose dependent way on L1210 cells and the detailed evidence including cell soma shrinkage, chromatin condensation and typical apotptotic DNA ladder pattern [60]. The cell apoptosis was also proven by the detection of changes on typical apoptosis related proteins such as Bcl-2 [38,77] and caspase 3/7 [24,61]. More apoptosis-like cell death were induced by **5** on K562 cell lines [38], and **1**, **7**, **10**, and **33** towards leukemia cells [24] (Table 6).

Though apoptosis-like cell death was concluded from most of the Ophs cytotoxicity mechanism studies, Morrison et al. (2016) found that compound **1** induced apoptosis only on five of eight tested cell lines, while the remaining three cell lines displayed necrosis (a form of traumatic cell death that results from acute cellular injury characterized by the loss of cell membrane integrity and an uncontrolled release of the products of cell death into the extracellular space), and only two rhabdomysosarcoma cell lines of the five apoptotic lines showed large increase of PARP cleavage caused by the caspase signaling pathway [64]. It means that not all the cell death induced by Ophs act in apoptosis mechanism, and even in apoptosis, the detailed pathway or target could be different.

It is difficult to draw a conclusion that Ophs cytotoxicity is based on an apoptosis mechanism, not only with the appearance of the necrosis, but also the paraptosis, which is an apoptosis-independent programmed cell death characterized by a process of vacuolization of the physical enlargement of mitochondria and endoplasmic reticulum that may be associated with the disruption of internal potassium ion homeostasis involving the big/large conductance Ca^+^-activated K^+^ channel (BKCa) [62]. Again, this finding was related to the Ca^+^ concentration increasing induced by **1**.

## 7. Anti-Influenza Activity

Small molecule **5** possessed the potential of anti-influenza activity against the influenza virus strain WSN on both in vitro and in vivo test. During in vitro experiment, when the A549 cells (infected with WSN virus) were treated with **5** at 0.5 μM, the influenza virus titers were reduced obviously, indicated that this molecule might inhibit influenza A virus replication through increasing the type III interferons and some interferon-stimulated genes. Additionally, at the same low concentration, the generation of A549 cells was not influenced. In vivo experiment, comparing with the infected control group, **5** decreased the death, pulmonary lesions, splenic atrophy, and thymic atrophy of mice infected with WSN at 0.3 mg/kg, intranasal inoculation [22].

## 8. Inflammation-Promoting Activity

It has been reported that inflammatory cytokines (IL-6, TNF-α), myeloperoxidase (MPO), heme oxygenase (HO), alanine aminotransferase (ALT), and aspartate aminotransferase (AST) play a pivotal role in oxidative stress and inflammatory processes. When treating male Wistar rats with **1** at 1.0 mg/kg (oral administration), the inflammatory mediators released rapidly through significantly elevating the concentrations of IL-6 and TNF-α as well as the activity of HO and MPO in plasma after 24 h of the administrations, which in fact promoted systemic inflammation. Comparing with the vehicle control group, the concentrations of IL-6 and TNF-α, as well as the activity of HO and MPO in the cardiac left ventricle of treatment group, did not change significantly, which indicated that treating **1** did not show toxic effects on the cardiac tissue and liver enzymes. However, though **1** induced inflammation in the plasma, the concentrations of ALT and AST did not change in both cardiac tissue and serum [23].

## 9. Other Activities

Apart from the activities discussed above, some other activities of Ophs were also reported. For example, compound **1** possesses antimalarial activity against *P. falciparum* at 1.3 μM [35].

Compound **23** induced hyperacusia in day-old chicks at the dose of 250 mg/kg while compound **16** induced no visible effects in day-old chicks at the dose of 375 mg/kg. Meanwhile, treating with **23** at the rates of 250 to 375 mg/kg, there was no mortality and the chicks fully recovered in 24 h [44]. Compounds **16**, **33**, and **34** exhibited inhibiting activity of factor X activated protein (factor Xa) with concentrations 155, 9.2, and 73 μM, respectively [78]. In addition, compounds **24** and **47** possessed the brine shrimp toxicity against *Artemia salina* with the LC_50_ values of 108 and 124 μM, respectively. Both led to lethality (>75%) at a higher concentration of 259 μM [43]. Furthermore, compound **10** competed the binding of the protein ^125^I-gp120 (an envelope protein, mediated the HIV-1 viral’s entrance into the cells) to human CCR5 at 40 μM [31].

## 10. Conclusions and Future Perspectives

Belonging to the tricarbocyclic sesterterpenoids with a 5-8-5 ring system structure, 49 natural Ophs have been reported and assigned into A–W in order of discovery. They display potential applications in various fields due to their broad spectrum of biological characteristics such as phytotoxic, antimicrobial, nematocidal, cytotoxic, anti-influenza, and inflammation-promoting activities. Particularly, these sesterterpenoid-type molecules exhibit anti-proliferative activities against a vast number of cancer cell lines mainly including breast, cervical, colon, glioblastoma multiforme (GBM), lung, leukemia, and melanoma carcinoma. They also display potent cytotoxic properties against multidrug resistance (MDR) and cancer stem cells (CSCs). The anti-proliferative activity against a broad range of cell lines entitle the sesterterpenoid-type Ophs with great possibilities in the utilization of their diverse pharmacological properties in drug development especially in cancer drug discovery. Actually, numerous drugs in clinical have been proven to exhibit broad biological activities. For example, recent investigation illustrated that clarithromycin, a well-known antibiotic drug, possessed anti-cancer effects in multiple tumor types on the base of its extensive preclinical and clinical data [79]. Indeed, the sesterterpenoid-type compounds are promising drug candidates, as current studies are attempting to prepare chemoembolization particles possessing the sustained release effects for the delivery of compound **1** to the target locations [63].

More studies are required for obtaining biological Ophs. Although many researchers have studied their chemical synthesis, merely few Ophs can be totally synthesized [80,81,82,83]. Traditional total synthesis of Ophs needs a complex multistep reaction. However, Brill et al. (2016) have discovered that 6-epi-ophiobolin-N could be totally synthesized in only nine steps, which is one of the shortest total synthesis steps for sesterterpenoids by far, through the “biomimetic synthetic strategy” [84]. However, there are very few reports on Ophs biosynthesis. Recent investigations demonstrate that the biosynthesis of Ophs was related to the first sesterterpene synthase (AcOS) [85] and the *oblB_Ac_* gene (cytochrome P450) from the cryptic gene cluster [86], and the biosynthesis of Ophs skeleton involves multiple gene clusters, which are in charge of C15, C20, C25, and C30 terpenoid biosynthesis [87].

Despite the vast number of investigations on biological and pharmacological properties of Ophs in the recent years, we still have only preliminary understanding of the structure–activity relationship and the functional mechanism of Ophs, which are crucial for uncovering novel molecular candidates. As discussed in this review, the structure–activity relationship is still not established. However, under many circumstances, it can be deduced that Ophs with a 6-alpha (6R) stereochemistry have weaker biological activities including phytotoxic, antibacterial, and nematocidal activities than Ophs with a 6S stereochemistry; Ophs with a tetrahydrofuran ring between C14 to C17 have stronger activities including phytotoxic, antifungal, and anticancer activities; Ophs with a tetrahydrofuran ring between C5 to C7 and C21 have significant weaker anti-CSC activities. Furthermore, the dicarbonyl at C5 and C21, and the hydroxyl group attached to C-3, C-6 or C14, are critical for Ophs’ anticancer activities.

The biological and pharmacological characteristics of Ophs are intrinsically associated with influencing the process of proliferation and mediating different pathways as a sure Ca^2+^/calmodulin antagonist. The bioactive molecules induce cell cycle arrest, apoptosis, paraptosis, and autophagic process by different mechanisms depending on the cell type, which revealed that Ophs acted on multiple targets. Additionally, the PE non-protein target related to lipid membrane may explain the broad bioactivities of Ophs on plants, microorganisms, nematode, trypanosome, and mammalian cells, since PE is ubiquitously found in the nature. Further investigations are needed to clarify whether this covalent reaction exists not only in membrane PE but also in the other proteins such as the calmodulin that possesses the primary amines. Furthermore, Additional research is needed in order to illustrate how this non-protein target connected with the other observations such as the pathways and the different morphologies of cell death such as apoptosis, paraptosis, and necrosis. More evidence is still required to elucidate whether Ophs are multi-targets compounds or a single target accounts for their biological activity, regardless of structure.

## Figures and Tables

**Table 1:** Sources of Ophs.

Number	Compound	Source	Reference	Source	Reference
**1**	Ophiobolin A	*Bipolaris oryzae*	[13,14,33]	*Drechslera oryzae* ^1^	[34]
		*Cochliobolus heterostrophus* ^2^	[30,35]	*Drechslera gigantea*	[12,28]
**2**	6-epi-ophiobolin A	*Bipolaris oryzae*	[13,14,33]	*Drechslera oryzae* ^1^	[34]
		*Penicillium patulum*	[30]	*Bipolaris* sp.	[36]
		*Cochliobolus heterostrophus* ^2^	[35]	*Drechslera gigantea*	[12,28]
**3**	3-anhydroophiobolin A	*Cochliobolus heterostrophus* ^2^	[30,35]	*Bipolaris oryzae*	[33]
		*Bipolaris setariae* NY1	[37]	-	-
**4**	3-anhydro-6-epi-ophiobolin A	*Bipolaris oryzae*	[13,14]	*Drechslera oryzae* ^1^	[34]
		*Bipolaris* sp.	[36]	*Cochliobolus heterostrophus* ^2^	[35]
		*Drechslera gigantea*	[12,28]	-	-
**5**	3-anhydro-6-hydroxy-Ophiobolin A	*Bipolaris oryzae*	[38]	-	-
**6**	Ophiobolin A lactone	*Polyangium cellulosum*	[30]	-	-
**7**	Ophiobolin B	*Helminthosporium oryzae* ^3^	[39]	*Drechslera oryzae* ^1^	[34]
		*Bipolaris oryzae*	[14]	*Cochliobolus heterostrophus* ^2^	[30]
		*Drechslera gigantea*	[28]	-	-
**8**	3-anhydro-6-epi-ophiobolin B	*Cochliobolus heterostrophus* ^2^	[35]	-	-
**9**	Ophiobolin B lactone	*Pseudomonas aeruginosa*	[30]	-	-
**10**	Ophiobolin C	*Bipolaris oryzae*	[13]	*Cochliobolus heterostrophus* ^2^	[18]
		*Mollisia* sp.	[31]	*Emericella variecolor* ^4^ GF10	[26]
		*Aspergillus insuetus*	[24,40]	*Aspergillus calidoustus*	[24,40]
**11**	6-epi-ophiobolin C	*Cochliobolus heterostrophus* ^2^	[18]	*Emericella variecolor* ^4^ GF10	[26]
**12**	18-dihydroophiobolin C	*Cochliobolus heterostrophus* ^2^	[18]	-	-
**13**	Ophiobolin D	*Cephalosporium caerulens*	[32]	-	-
**14**	Ophiobolin E	*Drechslera gigantea*	[28]	-	-
**15**	Ophiobolin F	*Cochliobolus heterostrophus* ^2^	[41,42]	*Aspergillus ustus* cf-42	[43]
**16**	Ophiobolin G	*Aspergillus ustus*	[44]	*Emericella variecolor* ^4^ GF10	[26]
		*Aspergillus* sp.	[7]	-	-
**17**	6-epi-ophiobolin G	*Emericella variecolor* ^4^ GF10	[26]	*Ulocladium* sp.	[29]
		*Aspergillus calidoustus*	[24,40]	*Emericella variecolor* ^4^	[27]
		*Aspergillus ustus* cf-42	[43]	-	-
**18**	(6a)-21,21-O-dihydroophiobolin G	*Aspergillus ustus*	[23,45]	*Ulocladium* sp.	[29]
**19**	(6a)-21-deoxyophiobolin G	*Aspergillus ustus* cf-42	[43]	-	-
**20**	3-anhydro-21-deoxyophiobolin G	*Aspergillus ustus*	[46]	-	-
**21**	(6a)-18,19,21,21-Otetrahydro-18,19-dihydroxy-ophiobolin G	*Aspergillus ustus*	[45]	-	-
**22**	(6a)-16,17-dihydro-21-deoxy-ophiobolin G	*Aspergillus* sp.	[7]	*Aspergillus ustus* cf-42	[42]
**23**	Ophiobolin H	*Aspergillus ustus*	[43,44,47]	*Cochliobolus heterostrophus* ^2^	[30]
		*Emericella variecolor*^4^ GF10	[26]	*Aspergillus insuetus*	[24,40]
**24**	(5a,6a)-ophiobolin H	*Aspergillus ustus*	[43,45]	-	-
**25**	5-O-methylophiobolin H	*Aspergillus ustus*	[45]	-	-
**26**	(5a,6a)-5-O-methylophiobolin H	*Aspergillus ustus*	[45]	-	-
**27**	Ophiobolin I	*Bipolaris oryzae*	[13,14,33]	*Drechslera oryzae* ^1^	[34]
		*Polyangium cellulosum* ^5^	[30]	*Bipolaris* sp.	[36]
		*Cochliobolus heterostrophus* ^2^	[35]	*Drechslera gigantea*	[12,28]
		*Bipolaris setariae* NY1	[37]	-	-
**28**	6-epi-ophiobolin I	*Drechslera oryzae* ^1^	[34]	-	-
**29**	25-Hydroxyophiobolin I	*Bipolaris oryzae*	[13]	*Drechslera oryzae* ^1^	[34]
**30**	Ophiobolin J	*Drechslera oryzae* ^1^	[34]	*Drechslera gigantea*	[28]
**31**	8-epi-ophiobolin J	*Drechslera gigantea*	[28]	-	-
**32**	8-deoxyophiobolin J	*Drechslera oryzae* ^1^	[34]	-	-
**33**	Ophiobolin K	*Aspergillus ustus*	[48]	*Cochliobolus heterostrophus* ^2^	[18]
		*Emericella variecolor* ^4^	[26,27]	*Aspergillus* sp.	[7,24,40]
		*Aspergillus insuetus*	[24,40]	*Aspergillus calidoustus*	[19,24,40]
**34**	6-epi-Ophiobolin K	*Aspergillus ustus*	[48]	*Cochliobolus heterostrophus* ^2^	[18]
		*Emericella variecolor* ^4^	[26,27]	*Aspergillus* sp.	[7,24,40]
		*Aspergillus insuetus*	[24,40]	*Ulocladium* sp.	[29]
		*Aspergillus calidoustus*	[19,24,40]	-	-
**35**	Ophiobolin L	*Cochliobolus heterostrophus* ^2^	[30]	-	-
**36**	6-epi-ophiobolin L	*Penicillium patulum* ^6^	[30]	-	-
**37**	Ophiobolin M	*Cochliobolus heterostrophus* ^2^	[49]	-	-
**38**	6-epi-ophiobolin M	*Cochliobolus heterostrophus* ^2^	[18]	-	-
**39**	6-epi-ophiobolin N	*Emericella variecolor* ^4^ GF10	[26]	*Aspergillus insuetus*	[24,40]
**40**	Ophiobolin O	*Aspergillus* sp.	[7]	-	-
**41**	6-epi-ophiobolin O	*Aspergillus* sp.	[7]	-	-
**42–46**	Ophiobolin P-T	*Ulocladium* sp.	[29]	-	-
**47–49**	Ophiobolin U-W	*Aspergillus insuetus*	[24,40]	*Aspergillus ustus*	[43]

The titles of columns 3 and 4 were the same status as columns 5 and 6, respectively. *Drechslera oryzae* ^1^, *Helminthosporium oryzae* ^3^ are reclassified in *Bipolaris oryzae*, Taxonomy ID: 101162; *Cochliobolus heterostrophus* ^2^ is reclassified in *Bipolaris maydis*, Taxonomy ID: 5016; *Emericella variecolor* ^4^ is reclassified in *Aspergillus stellatus*, Taxonomy ID: 1549217; *Polyangium cellulosum* ^5^ is reclassified in *Sorangium cellulosum*, Taxonomy ID: 56; *Penicillium patulum* ^6^ is reclassified in *Penicillium griseofulvum*, Taxonomy ID: 5078.

**Table 2:** Phytotoxic activities of Ophs by the leaf-wounding assay.

Plant	1	2	3	4	7	10	27	29
Barley	>4.5 ^a^	3.0–4.5 ^a^	3.0–4.5 ^a^	<0.5 ^a^	0.5–1.5 ^a^	NA	0.5–1.5 ^a^	NA
Cabbage	0–2 *^, b^	>2 *^, b^	>0 *^, b^	>0 *^, b^	>0 *^, b^	NA	>0 *^, b^	NA
Cocklebur	0.5–1.5 ^a^	1.5–3.0 ^a^	1.5–3.0 ^a^	<0.5 ^a^	0.5–1.5 ^a^	NA	0.5–1.5 ^a^	NA
Corn	3.0–4.5 ^a^	1.5–3.0 ^a^	3.0–4.5 ^a^	0.5–1.5 ^a^	0.5–1.5 ^a^	NA	0.5–1.5 ^a^	NA
	>2 *^, b^	0–2 *^, b^	>2 *^, b^	>0 *^, b^	>0 *^, b^	NA	N *^, b^	NA
	2–3 ^c^	>3 ^c^	NA	NA	0.5–2.0 ^c^	0.5–2.0 ^c^	0.5–2.0 ^c^	0.5–2.0 ^c^
Goose grass	1.5–3.0 ^a^	3.0–4.5 ^a^	1.5–3.0 ^a^	1.5–3.0 ^a^	1.5–3.0 ^a^	NA	1.5–3.0 ^a^	NA
Grain sorghum	3.0–4.5 ^a^	1.5–3.0 ^a^	3.0–4.5 ^a^	1.5–3.0 ^a^	3.0–4.5 ^a^	NA	0.5–1.5 ^a^	NA
Johnson grass	2–3 ^c^	>3 ^c^	NA	NA	0.5–2.0 ^c^	0.5–2.0 ^c^	0.5–2.0 ^c^	0.5–2.0 ^c^
Rice	3.0–4.5 ^a^	1.5–3.0 ^a^	3.0–4.5 ^a^	0.5–1.5 ^a^	0.5–1.5 ^a^	NA	0.5–1.5 ^a^	3.0–4.5 ^a^
	>0 *^, b^	0–2 *^, b^	0–2 *^, b^	>0 *^, b^	0–2 *^, b^	NA	>0 *^, b^	NA
Sorghum	2–3 ^c^	2–3 ^c^	NA	NA	0.5–2.0 ^c^	0.5–2.0 ^c^	0.5–2.0 ^c^	0.5–2.0 ^c^
Soybean	1.5–3.0 ^a^	1.5–3.0 ^a^	1.5–3.0 ^a^	<0.5 ^a^	1.5–3.0 ^a^	NA	0.5–1.5 ^a^	NA
Tobacco	>0 *^, b^	>0 *^, b^	>0 *^, b^	N ^b^	>0 *^, b^	NA	>0 *^, b^	NA
Velvet-leaf	0.5–1.5 ^a^	<0.5 ^a^	1.5–3.0 ^a^	<0.5 ^a^	<0.5 ^a^	NA	0.5–1.5 ^a^	NA

The phytotoxic activities are characterized by necrosis diameter, mm; * at the concentration of 1.2 × 10^3^ μM; other unmarked, 1.0 × 10^3^ μM; N, no phytotoxic activity; NA, not available; ^a, b, c,^ refer to References [15], [14], [13], respectively.

**Table 3:** Antibacterial activities of Ophs.

Number	Antibacterial Activity						
	E. coli *	S. aureus *	MRSA	*M. intracellulare*	*B*. *subtilis*	BCG	*M. smegmatis*
**1**	>62 ^a^	62/7–12 ^a^	NA	62/3–6 ^a^	NA	NA	NA
**3,4**	NA	>100 ^b^	>100 ^b^	NA	>100 ^b^	>100 ^b^	NA
**5**	NA	31 ^b^	31 ^b^	NA	31 ^b^	31 ^b^	NA
**7**	>62 ^a^	62/3–6 ^a^	NA	>62 ^a^	NA	NA	NA
**16**	>2.7 × 10^3 c^	NA	NA	NA	136/10 ^c^	NA	NA
**17**	NA	NA	>250^d^	NA	>250 ^d^	125/NR ^d^	68/NR ^e^
**18**	NA	NA	>250 ^d^	NA	>250 ^d^	125/NR ^d^	NA
**23**	>2.6 × 10^3 c^	>1.6 × 10^3 f^	NA	NA	682/16 ^c^	NA	NA
**24**	1.6 × 10^3^/10 ^f^	>1.6 × 10^3 f^	NA	NA	NA	NA	NA
**27**	>63 ^a^	>63 ^a^	NA	63/3–6 ^a^	NA	NA	NA
**33**	NA	NA	NA	NA	NA	64/NR ^e^	33/NR ^e^
**34**	NA	NA	125/NR ^d^	NA	125/NR ^d^	>250 ^d^	260/NR ^e^
**35**	>60.^a^	>60 ^a^	NA	60/3–6 ^a^	NA	NA	NA
**36**	>60 ^a^	60/>12 ^a^	NA	>60 ^a^	NA	NA	NA
**42**	NA	NA	31/NR ^d^	NA	62/NR ^d^	>250 ^d^	NA
**43–45**	NA	NA	>250 ^d^	NA	>250 ^d^	>250 ^d^	NA
**46**	NA	NA	16/NR ^d^	NA	31/NR ^d^	31/NR ^d^	NA
**47**	1.6 × 10^3^/15 ^f^	1.6 × 10^3^/10 ^f^	NA	NA	NA	NA	NA

The antibacterial activities are assessed on concentration/inhibitory diameter, μM/mm; * indicate compound **12**, **16**, **18**, **44** and **45** showed no activity against the pathogen at 1.7, 1.8, 1.7, 1.6, 1.6 × 10^3^ μM, respectively; *E. coli*: *Escherichia coli*; *S. aureus: Staphylococcus aureus; M. intracellulare: Mycobacterium intracellulare; B. subtilis: Bacillus subtilis;* MRSA: methicillin-resistant *Staphylococcus aureus*; BCG: *Bacille* Calmette-Guerin; *M. smegmatis: Mycobacterium smegmatis.* NA, not available; NR, using MIC, no reported inhibitory diameter; ^a, b, c, d, e, f,^ refer to references [30], [38], [44], [29], [27], and [43], respectively.

**Table 4:** Nematocidal activity of Ophs.

Number	Species	Concentration (LD_50_, μM)	Reference
**10**	*C*. *elegans*	5	[18]
**11**	*C*. *elegans*	130	[18]
**12**	*C*. *elegans*	25	[18]
**33**	*C*. *elegans*	26	[18]
	*T. cruzi*	13	[19]
**34**	*C*. *elegans*	>260	[18]
	*T. cruzi*	9.6	[19]
**37**	*C*. *elegans*	13	[18]
**38**	*C*. *elegans*	130	[18]

**Table 5:** Cytotoxic activities of Ophs.

Number	Cell Line	IC_50_ (μM)	Reference	Cell Line	IC_50_ (μM)	Reference
**1**	CLL	1 × 10^−3^ *	[24]	P388	0.06	[35]
	HUVECs	0.08	[58]	MNA	<0.12	[59]
	HT-29	0.12	[35]	A549	0.12, 0.42 ± 0.01	[35,58]
	FFL	0.12	[59]	PK-15	0.12–0.24	[59]
	OVCAR3	0.28	[58]	B16F10	0.29 ± 0.05	[21]
	L1210	0.3	[60]	SKMEL28	0.37 ± 0.03	[21]
	RPMI8226	0.4	[61]	Hs683	0.62 ± 0.04	[21]
	MM1R	0.7	[61]	U266B1	0.7	[61]
	U373-MG	0.87 ± 0.01	[62]	Jurkat	1.0	[61]
	A2780	1.2	[61]	RD	1–2, 1.3	[63,64]
	KB 3-1	1.4	[64]	RH30	1.5	[64]
	MDAMB-231	1.9, 0.7	[61,64]	T98G	1.9 ± 0.2	[62]
	PC3	2.5	[61]	U2OS	2.8	[64]
	GL19	3.7 ± 1.4	[62]	U-87MG	3.8	[64]
	MCF-7	4.3; 4.0	[61,64]	LLC-GA5-COL150	7.9 ± 0.40	[65]
	hPBMC	21	[61]	HeLa	62; 4.5	[33,64]
	KB	78	[33]	-	-	-
**2**	HeLa	14	[33]	KB	22	[33]
**3**	HeLa	10	[33]	KB	12	[33]
	K562	40 ± 5.5	[38]	HepG 2	56 ± 3.8	[38]
	CLL	Inactive *	[24]	-	-	-
**4**	B16F10	22 ± 3	[21]	SKMEL28	27 ± 0.4	[21]
	A549	30 ± 1	[21]	Hs683	30 ± 3	[21]
	K562	36 ± 4.4	[38]	HepG 2	47 ± 4.8	[38]
	CLL	Inactive *	[24]	-	-	-
**5**	K562	4.1 ± 0.50	[38]	HepG2	6.5 ± 0.34	[38]
**7**	CLL	2 × 10^−3^ *	[24]	-	-	-
**10**	CLL	8 × 10^−3^ *	[24]	-	-	-
**16**	P388	25	[7]	-	-	-
**17**	HepG2	0.37 ± 0.03	[29]	KB	1.4 ± 0.06	[29]
	CLL	Inactive *	[24]	-	-	
**18**	HepG2	2.2 ± 0.18	[29]	KB	4.0 ± 0.22	[29]
	L5178Y	>27	[45]	-	-	-
**21**	L5178Y	>25	[45]	-	-	-
**23**	P388	106	[7]	CLL	Inactive *	[24]
**24–26**	L5178Y	>25	[45]	-	-	-
**7**	HeLa	0.25	[33]	HUVECs	>1	[58]
	KB	2.3	[33]	OVCAR3	71	[58]
**33**	CLL	4 × 10^−3^ *	[24]	ACHN	0.27	[26]
	HCT116	0.33	[26]	T-47D	0.35	[26]
	P388/ADR	0.36	[26]	TK-10	0.51	[19]
	MCF-7	0.51	[19]	P388	0.51; 13	[7,26]
	MDA-MB-231	0.57	[26]	NCI-H460	0.57	[26]
	HOP18	0.65	[26]	-	-	-
**34**	HepG2	1.9 ± 0.11	[29]	TK-10	3	[19]
	MCF-7	3	[19]	KB	4.7 ± 0.72	[29]
	P388	25	[7]	CLL	Inactive *	[24]
**39**	CLL	Inactive *	[24]	-	-	-
**40**	P388	4.7	[7]	MCF-7	13 ± 1.3	[66]
	HUVEC	85 ± 12	[66]	DU145	16 ± 2.7	[66]
	NCI-H460	14 ± 1.0	[66]	-	-	-
**41**	P388	9.3	[7]	-	-	-
**42**	HepG2	1.5 ± 0.10	[29]	KB	6.2 ± 0.25	[29]
**43**	HepG2	1.3 ± 0.12	[29]	KB	2.4 ± 0.21	[29]
**44**	HepG2	1.2 ± 0.08	[29]	KB	2.9 ± 0.28	[29]
**45**	HepG2	1.4 ± 0.08	[29]	KB	4.9 ± 0.32	[29]
**46**	HepG2	0.24 ± 0.02	[29]	KB	3.0 ± 0.24	[29]

The cytotoxic activities of Ophs with the cell lines in ascending order of the IC_50_ values by MTT assay; * LC_50_ value, cytotoxic activities test by CellTiter-Glo^®^ assay; (1) Human cancer cell lines: brain cancer cells, Hs683; breast carcinoma cells, MCF-7, MDA-MB-231 and T-47D; cervical carcinoma cells, HeLa; colon carcinoma cells, HCT116 and HT-29; GBM cells, GL19, U373-MG, T98G and U-87MG; haematological cancer cells, Jurkat, MM1R, RPMI8226, U266B1; hepatocarcinoma cells, HepG2; large cell lung carcinoma cells, NCI-H460; leukemia cells, CLL, K562 and L1210; lung carcinoma cells, A549, NCI-H460 and HOP18; melanoma cells, B16F10 and SKMEL28; nasopharyngeal carcinoma cells, KB and KB 3-1; osteosarcoma cells, U2OS; ovarian cancer cells A2780 and OVCAR3; prostate cancer cells, DU145 and PC3; renal carcinoma cells, ACHN; rhabdomyosarcoma cancer cells, RD and RH30; (2) Human normal cell lines: hPBMC, peripheral blood mononuclear cells; HUVEC, human umbilical vein endothelial cell line; (3) Other mammalian cell lines: adriamycin resistant cells, P388/ADR; feline fetus lung cells, FFL; mouse leukemia cells, P388; murine lymphoma cells, L5178Y; murine neuroblastoma cells, MNA; porcine kidney cells, PK-15; (4) LLC-GA5-COL150 cells, cells transfected with human multidrug resistance 1 (MDR1) cDNA encoding P-gp.

**Table 6:** Cell death induced by ophiobolins.

Cell Death	Observations	Ophs	Cell Lines	Reference
Apoptosis	Cell soma shrinkage, chromatin condensation, typical apotptotic DNA ladder	**1**	L1210	[60]
	Changes of caspase 3/7	**1**	MDA-MB-231	[61]
	* AV^+^/PI^−^, AV^+^/PI^+^ and PARP increase	**1**	RD, RH30	[64]
	AV^+^/PI^+^ increase	**1**	MDA-MB-231, MCF-7 HeLa	[64]
	AV^+^, Caspase-3 activation	**1, 7, 10, 33**	CLL	[24]
	Bcl-2 decrease	**5**	K562	[38]
	Bcl-2 decrease	**40**	MCF-7	[77]
Necrosis	AV^−^/PI^+^ increase	**1**	KB 3-1, U2OS, U-87 MG	[64]
Paraptosis	BKCa decreasing, cytoplasmic vacuolization and mitochondrial swelling	**1**	U373-MG, GL19	[62]

* Annexin V binds phosphatidyl serine and can be used as a probe for cell death via an apoptotic pathway. Combination of Annexin V and PI (which stains the DNA of cells with a permeable membrane, i.e., necrotic cells) can determine early apoptotic cells which are Annexin V positive and PI negative (AV^+^/PI^−^) [64].
